# Reduced risk of dementia with recombinant zoster vaccine in US adults age 65 or older

**DOI:** 10.1002/alz.71407

**Published:** 2026-04-28

**Authors:** Susan dos Reis, Phuong Tran, Kareshma Mohanty, Alejandro Amill‐Rosario, Abree Johnson, Kathleen Ryan, Hannah Alsdurf, Driss Oraichi, Huifeng Yun

**Affiliations:** ^1^ Department of Practice, Sciences, and Health Outcomes Research University of Maryland School of Pharmacy Baltimore Maryland USA; ^2^ Global Vaccine and Infectious Disease Epidemiology, GSK Rockville Maryland USA

**Keywords:** Alzheimer's disease, dementia, Medicare, recombinant zoster vaccine

## Abstract

**INTRODUCTION:**

Herpes zoster vaccines may lower the risk of dementia onset. We evaluated new‐onset dementia among US Medicare beneficiaries ≥65 years old following recombinant zoster vaccination (RZV).

**METHODS:**

We matched one RZV‐exposed to two RZV‐unvaccinated on age, sex, and race/ethnicity. Individuals were ≥65 years old on the RZV dose 2 date (RZV‐exposed) or preventive care visit date (RZV‐unvaccinated), enrolled in Medicare ≥11 months before this date, and had no pre‐existing dementia of any type. Weighted Cox proportional hazards models generated hazard ratios (HR) of new‐onset dementia, Alzheimer's disease (AD), and vascular dementia (VD).

**RESULTS:**

The incidence per 1000 person‐years of new‐onset dementia for 15,061/502,845 RZV‐exposed and 36,526/1,005,690 RZV‐unvaccinated was 10.45 and 15.73, respectively. Time‐dependent HRs (95% confidence interval) for ≤3 and >3 years’ follow‐up were as follows: 0.67 (0.65, 0.68); 0.74 (0.69, 0.79) for dementia; 0.72 (0.69, 0.74); 0.83 (0.74, 0.94) for AD; 0.67 (0.64, 0.70); 0.66 (0.57, 0.78) for VD.

**DISCUSSION:**

Two‐dose RZV may lower new‐onset dementia, AD, and VD risk.

## Highlights

- RZV reduces dementia risk in US adults age 65 or older.
- There was a lower risk of AD with RZV.
- The risk of VD was lower with RZV.
- A 3‐ and 6‐month induction period corroborated the lower risk from the main results.

## 1 BACKGROUND

Dementia, a syndrome associated with a general cognitive decline stemming from neurodegeneration, is projected to increase from 50 to 152 million people globally by 2050.^1^, ^2^ Among adults worldwide aged 65 and older, 8.1% have dementia.^3^ In 2020, 6.1 million Americans were living with Alzheimer's disease (AD), the most common type of dementia.^4^ Vascular dementia (VD), which is due to vascular brain injury, is the next most common type of dementia.^5^ While the etiology of dementia is unclear, infections such as herpes zoster (HZ) may increase the risk of developing dementia, but evidence has been inconclusive. Some studies suggest a strong,^6^, ^7^ mild,^8^ or no association.^9^ Several population‐based studies of adults aged 65 years or older using administrative data representing US veterans and US citizens with private health insurance suggest a lower risk of dementia following HZ vaccination. Comparing HZ vaccination with no vaccination, the hazard ratios (HR) and 95% confidence intervals (CIs) were 0.69 (95% CI: 0.67, 0.72) and 0.65 (95% CI: 0.57, 0.74) for US veterans and citizens with private health insurance, respectively.^10^ Another study also found a lower risk of dementia after HZ vaccination, with HR = 0.75 (95% CI: 0.71, 0.79) for US veterans and HR = 0.67 (95% CI: 0.57, 0.80) for US adults with commercial insurance plans.^11^ Similar risk estimates were reported in studies conducted in the United Kingdom (UK) and Wales.^12^, ^13^, ^14^ This prior research included all HZ vaccines without distinguishing the effect of a single product.

A few recent studies investigated the risk of dementia specifically following vaccination with recombinant zoster vaccine (RZV), a two‐dose series vaccine that was approved by the US Food and Drug Administration (FDA) in October 2017 for HZ prevention in adults ≥50 years old. Data from US adults aged ≥50 years reported a significantly lower risk of dementia for the two‐dose relative to a single RZV dose.^15^ Research on older US adults in a Medicare Advantage healthcare coverage plan provides further evidence for RZV and reduction in the risk of AD.^16^ Taquet et al.^17^ compared adults aged ≥65 years who received the live shingles vaccine prior to November 2017, that is, before the market approval of RZV, with those who received RZV in or after November 2017, that is, after market approval. The authors reported a statistically significant lower risk of dementia that translated into an average of 164 days diagnosis free.^17^

**RESEARCH IN CONTEXT**

1. **Systematic review**: The authors conducted a comprehensive review using PubMed to identify relevant evidence related to HZ vaccine and dementia risk. Population‐based studies of adults aged ≥65 years old demonstrated a lower risk of dementia, AD, and VD following HZ vaccination, which is consistent with findings reported from the UK.
2. **Interpretation**: We show in a representative Medicare population of US adults aged ≥65 years old that RZV reduces the risk of dementia, AD, and VD. Our study contributes evidence to bolster the generalizability of the findings across different populations.
3. **Future directions**: The manuscript provides evidence that RZV may reduce the risk of new‐onset dementia, including AD and VD. Further studies on the mechanism of action and the impact of delayed onset of dementia on individual outcomes would help to better understand the clinical and public health impact.

Two recently published studies employing a quasi‐experimental design demonstrated reduction in the probability of dementia after a HZ vaccine program roll‐out based on regression discontinuity percentage points.^18^, ^19^ In these analyses, no distinction was made between the live zoster vaccine and RZV or they did not include RZV because it had not yet been approved.^18^, ^19^ While the works of Eyting et al.^18^ and Pomirchy et al.^19^ represent important contributions to the field, the authors call for replication of this research in different populations and settings. It is important to note the live zoster vaccine is no longer available in the United States, so the current study constitutes a valuable contribution and can advance knowledge of the potential effects of RZV on the reduction of the risk of dementia.

Most studies used RZV‐unvaccinated or historical controls and did not implement a concurrent comparator arm. Since RZV is a routine preventive vaccine, comparators drawn from those who are RZV‐unvaccinated at the time of a routine preventive care visit would be appropriate and would aid in mitigating healthy‐user bias. Further, while overall dementia covers all types, including AD and VD, there was insufficient evidence to know whether the effect of HZ vaccination was specific to a subtype of dementia. Therefore, this study aimed to assess the risk of new‐onset dementia and AD and VD dementia subtypes among US Medicare beneficiaries aged ≥65 years who had received the two‐dose RZV regimen relative to RZV‐unvaccinated comparators who had had a preventive care visit but had not yet received the RZV vaccine.

## 2 METHODS

The University of Maryland Baltimore Institutional Review Board approved the study. This study followed the Strengthening the Reporting of Observational Studies in Epidemiology (STROBE) reporting guidelines.

### 2.1 Data source

We used Centers for Medicare and Medicaid Services (CMS) Chronic Conditions Warehouse (CCW) Medicare administrative data for a 20% random sample of US adults enrolled in Medicare (https://www2.ccwdata.org/web/guest/home/). The CCW data include inpatient, outpatient, physician, and prescription claims for all health services and medications provided for Medicare enrollees with fee‐for‐service Parts A, B, and D insurance coverage.

### 2.2 Study design

This study was a comparator cohort design. Receipt of the second RZV dose, any time in the calendar years 2018 to 2020, defined the RZV‐exposed. The National Drug Code on a pharmacy claim or the procedure code 90750 on a Medicare Part B physician office setting claim determined RZV exposure. We identified RZV‐unvaccinated comparators from those who had at least one preventive care visit at any time in the calendar years 2018 to 2020 and had not received RZV prior to or on the preventive care visit date. To maximize the comparability of the groups and minimize confounding, we implemented time‐based exposure sets, matching two eligible RZV‐unvaccinated comparators to one RZV‐exposed on age (within 2‐year bands), sex, and race/ethnicity within 3‐month calendar intervals, relative to the RZV dose 2 date. Since individuals could have multiple preventive care visits in the 3‐month interval, we randomly selected one preventive care visit date and removed individuals with unknown race prior to matching.

The index date was the RZV dose 2 date for RZV‐exposed and the preventive care visit date for the matched RZV‐unvaccinated comparator. The follow‐up period started on day 1 after the index date and continued until the earliest of the outcome of interest or a censoring event, that is, end of the study period (i.e., December 31, 2022); disenrollment from fee‐for‐service Medicare; death; receipt of zoster live vaccine (ZVL); or receipt of RZV (for the RZV‐unvaccinated comparators). Medical comorbidities, medication use, health service utilization, and preventive vaccines (i.e., influenza and pneumococcal) were identified in the 365‐day baseline period preceding and including the index date. The study design is illustrated in Figure S1.

### 2.3 Study population

US Medicare beneficiaries who met all the following inclusion criteria were eligible for the analytic cohort: (1) receipt of two doses of RZV for the RZV‐exposed and at least one preventive care visit with no prior RZV for the RZV‐unvaccinated comparator; (2) ≥65 years old on the index date; (3) continuously enrolled in fee‐for‐service Medicare Parts A, B, and D for at least 11 of the 12 months preceding the index date. The list of preventive care visits is provided in Table S1.

We excluded those with any enrollment in Medicare Part C (i.e., Medicare Advantage) in the 365 days preceding the index date. We removed individuals with Medicare Part C because medical encounters are paid through a Medicare Advantage third‐party health plan, and these claims are not available in the CMS CCW data. Those with evidence of any dementia (including Lewy body dementia), VD, AD, mild cognitive impairment, or age‐related cognitive decline in all available data from 2017 up to and including the index date were excluded. The study leveraged the CCW variables that record the date of the first ever AD and related disorders, senile dementia, or non‐AD dementia to exclude any prevalent dementia.

### 2.4 Outcome measurement

The primary outcome was new‐onset dementia, including any type of dementia. Secondary outcomes were new‐onset AD and VD. New onset required individuals to have no evidence of dementia, AD, or VD prior to the index date. Outcome ascertainment was based on an International Classification of Diseases 10th Revision (ICD‐10) diagnosis code on one inpatient or two outpatient claims at least 7 days apart. We used a claims‐based dementia case identification algorithm that demonstrated 93% to 98% specificity and 31% to 79% sensitivity.^20^, ^21^ We did not impose a restriction on the timing of the second claim to preserve the integrity of the algorithm. The same algorithm with a 3‐year observation period had a specificity of 95% and sensitivity of 49%.^21^ To distinguish AD, VD, and other dementias, we implemented the codes provided by CMS in their guidance documentation. The ICD‐10 diagnosis codes and case ascertainment algorithms are presented in Table S2.

### 2.5 Covariates

The covariates were derived from a comprehensive and targeted review of the published literature on dementia risk factors and the association between HZ vaccine and the risk of dementia onset. Covariates included medical comorbidities, prescription medications, healthcare utilization, and certain preventive vaccinations. We used the ICD‐10 diagnosis and Healthcare Common Procedure Coding System (HCPCS) procedure codes in any position on inpatient, outpatient, and physician billing claims to identify the covariates. A description of the covariates and collection timeframe is given in Table S3. Most covariates were measured in the 365‐day baseline period preceding the index date. All available data from 2017 up to the index date were used to identify ZVL and tetanus, diphtheria, and pertussis (Tdap) vaccinations.

### 2.6 Statistical analysis

We used propensity scores (PS) with inverse probability of treatment weighting (IPTW) to adjust for measured confounding. The PS was estimated using a logistic regression model as the probability of receiving RZV dose 2 given the baseline covariates. The PS distribution for the RZV‐exposed and the RZV‐unvaccinated comparator was graphically displayed to assess the overlap and presence of extreme weights. We multiplied the IPTW by the treatment prevalence to minimize extreme weights.^22^, ^23^ The standardized mean difference (SMD) assessed imbalance in covariates between RZV‐exposed and RZV‐unvaccinated comparators. Absolute SMD less than 0.10 was indicative of good balance.^22^

The incidence rate per 1000 person‐years and the cumulative incidence were estimated for the primary and secondary outcomes. We implemented an IPTW‐weighted cause‐specific Cox proportional hazards regression model. We used the Aalen‐Johansen estimator to adjust the cumulative incidence function for death as a competing risk.^24^ A robust sandwich covariance matrix was used to estimate HRs and 95% CIs. We evaluated violations of the proportional hazard assumption by plotting the log[−logS(t)] versus log(t) and by testing the interaction between the exposure and log(t). Violation of the proportional hazards assumption was addressed by including a time‐dependent treatment coefficient, based on the time‐treatment interaction, as a constant in the weighted Cox model.^25^, [Fig alz71407-fig-0001]

We conducted several sensitivity analyses to test for potential biases that could distort the effect estimate. A subsample of RZV‐exposed with 2 to 6 months between dose administration per the FDA label and their matched RZV‐unvaccinated comparators evaluated the primary outcome of dementia. The potential bias given the long latency of dementia onset was addressed by imposing a 90‐day and a 183‐day induction period at the start of follow‐up. We excluded those who disenrolled from fee‐for‐service Medicare Parts A, B, and D or died in the induction period. Sensitivity analyses addressed the potential confounding due to prior Tdap and ZVL exposure, both of which have been shown to reduce the risk of dementia onset.^16^ Since sample size restrictions prohibited a separate Tdap matched comparator, we restricted the cohort to RZV‐exposed and RZV‐unvaccinated comparators who received Tdap prior to the index date. To address potential confounding by prior ZVL, we limited the cohort to those without ZVL exposure prior to the index date. Finally, we conducted separate analyses within age (i.e., 65 to 69, 70 to 74, 75 to 79, 80 to 84, 85+) and sex strata to explore the stratum‐level risk of dementia, the primary outcome, among RZV‐exposed relative to the RZV‐unvaccinated comparator.

We conducted a quantitative bias analysis using the E‐value to evaluate the potential for unmeasured confounding to explain away the effect estimate.^26^ Statistical significance was determined by the 95% CIs that did not include the null. All analyses were conducted using SAS (Statistical Analysis System 9.4, Carey, NC, USA).

## 3 RESULTS

### 3.1 Cohort characteristics

**FIGURE 1 alz71407-fig-0001:**
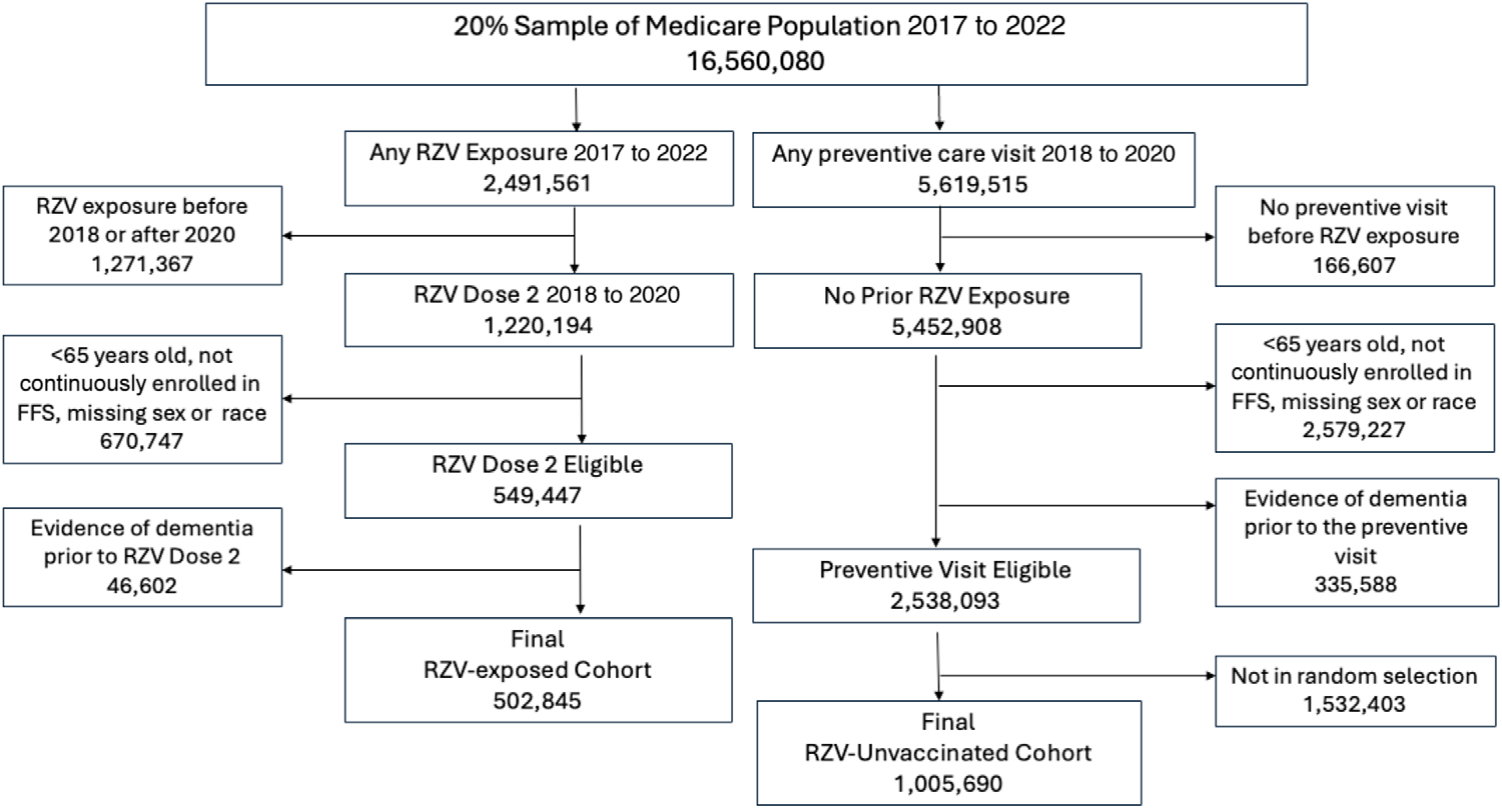
Flow diagram of cohort selection. RZV, recombinant zoster vaccine; FFS, fee for service.

The analytic cohort was composed of 502,845 RZV‐exposed and 1,005,690 RZV‐unvaccinated comparators (Figure 1). Both the RZV‐exposed and RZV‐unvaccinated comparators were 60% female, 93% White, and 55% aged 70 to 79 years old. The most prevalent medical condition was cardiovascular disease, with over 80% in each group, followed by diabetes and obesity (both >24%), and chronic kidney disease (>20%). A similar proportion had cancer or received chemotherapy. Mental health and substance use disorders were less common. Over 80% had a medication to treat cardiovascular disease and over a third received a psychotropic medication. The unweighted and IPTW‐stabilized weighted characteristics are presented in Table 1.

**TABLE 1:** Baseline demographic and health characteristics of RZV‐exposed and RZV‐unvaccinated comparators, before and after IPTW.

	Unweighted			IPTW‐stabilized		
	RZV‐exposed (*N *= 502,845)	RZV‐Unvaccinated (*N * = 1,005,690)		RZV‐exposed (*N * = 499,416)	RZV‐Unvaccinated (*N * = 1,007,340)	
	*N * (%)	*N * (%)	ASMD	*N * (%)	*N * (%)	ASMD
**Demographics**						
Age at index date, years						
Mean (SD)	73.95 (6.05)	73.97 (6.12)	0.0028	73.95 (6.01)	73.95 (6.11)	0.0003
Median (IQR)	73 (69 to 78)	73 (69 to 78)		73 (69 to 78)	73 (69 to 78)	
Age at index date, years						
65 to 69	137,230 (27.29)	275,907 (27.43)	0.0032	136,322 (27.3)	275,827 (27.38)	0.0019
70 to 74	161,882 (32.19)	322,317 (32.05)	0.0031	161,018 (32.24)	324,158 (32.18)	0.0013
75 to 79	113,024 (22.48)	226,261 (22.50)	0.0005	112,474 (22.52)	226,759 (22.51)	0.0003
80 to 84	58,267 (11.59)	116,321 (11.57)	0.0007	57,776 (11.57)	116,227 (11.54)	0.0010
85+	32,442 (6.45)	64,884 (6.45)	0.0000	31,824 (6.37)	64,369 (6.39)	0.0007
Sex						
Male	203,577 (40.49)	407,154 (40.49)	0.0000	195,814 (39.21)	403,550 (40.06)	0.0174
Female	299,268 (59.51)	598,536 (59.51)	0.0000	303,601 (60.79)	603,791 (59.94)	0.0174
Race/ethnicity^a^						
Asian	10,853 (2.16)	21,706 (2.16)	0.0000	10,845 (2.17)	21,739 (2.16)	0.0009
Black or African American	11,474 (2.28)	22,948 (2.28)	0.0000	11,056 (2.21)	22,682 (2.25)	0.0026
Hispanic	2664 (0.53)	5328 (0.53)	0.0000	2546 (0.51)	5246 (0.52)	0.0015
Other^b^	11,070 (2.20)	22,140 (2.20)	0.0000	11,042 (2.21)	22,194 (2.2)	0.0005
White	466,784 (92.83)	933,568 (92.83)	0.0000	463,927 (92.89)	935,480 (92.87)	0.0011
US region						
Northeast	98,735 (19.64)	203,649 (20.25)	0.0154	100,061 (20.04)	201,786 (20.03)	0.0001
Midwest	124,982 (24.85)	236,749 (23.54)	0.0307	121,523 (24.33)	242,159 (24.04)	0.0069
South	166,554 (33.12)	381,742 (37.96)	0.1012	179,618 (35.97)	365,279 (36.26)	0.0062
West	112,326 (22.34)	182,580 (18.15)	0.1042	97,874 (19.60)	197,329 (19.59)	0.0002
Other	242 (0.05)	954 (0.09)	0.0175	339 (0.07)	788 (0.08)	0.0038
Index date						
2018	96,402 (19.17)	192,804 (19.17)	0.0000	97,247 (19.47)	193,341 (19.19)	0.0071
2019	223,408 (44.43)	446,816 (44.43)	0.0000	222,802 (44.61)	448,287 (44.5)	0.0022
2020	183,035 (36.40)	366,070 (36.40)	0.0000	179,367 (35.92)	365,712 (36.3)	0.0081
**Chronic medical conditions**						
Autoimmune conditions						
Colitis	5038 (1.00)	9372 (0.93)	0.0072	4857 (0.97)	9683 (0.96)	0.0011
Crohn's disease	2808 (0.56)	5323 (0.53)	0.0040	2743 (0.55)	5473 (0.54)	0.0008
Fibromyalgia	8489 (1.69)	20,431 (2.03)	0.0254	9884 (1.98)	19,405 (1.93)	0.0038
Graves’ disease	1853 (0.37)	3566 (0.35)	0.0023	1826 (0.37)	3631 (0.36)	0.0009
Lupus	2002 (0.40)	4839 (0.48)	0.0125	2286 (0.46)	4593 (0.46)	0.0003
Multiple sclerosis	1571 (0.31)	3263 (0.32)	0.0021	1604 (0.32)	3229 (0.32)	0.0001
Psoriasis/psoriatic arthritis	11,509 (2.29)	21,428 (2.13)	0.0108	11,096 (2.22)	22,092 (2.19)	0.0020
Rheumatoid arthritis	16,108 (3.20)	36,587 (3.64)	0.0239	17,733 (3.55)	35,264 (3.50)	0.0027
Vasculitis	553 (0.11)	1097 (0.11)	0.0003	576 (0.12)	1114 (0.11)	0.0014
Cancer/chemotherapy	113,627 (22.60)	217,067 (21.58)	0.0244	110,098 (22.05)	221,067 (21.95)	0.0024
Cardiovascular diseases (any)	450,866 (89.66)	921,322 (91.61)	0.0669	454,043 (90.91)	917,567 (91.09)	0.0061
Congestive heart failure	32,315 (6.43)	85,927 (8.54)	0.0805	38,806 (7.77)	78,468 (7.79)	0.0007
Hypertension	351,461 (69.89)	751,873 (74.76)	0.1090	364,746 (73.03)	735,760 (73.04)	0.0001
Ischemic heart disease/MI	104,339 (20.75)	233,609 (23.23)	0.0599	110,701 (22.17)	224,582 (22.29)	0.0031
Other cardiovascular	402,215 (79.99)	814,396 (80.98)	0.0250	404,125 (80.92)	812,942 (80.70)	0.0055
Cerebrovascular condition	28,036 (5.58)	64,309 (6.39)	0.0345	30,254 (6.06)	61,542 (6.11)	0.0022
Cerebral infarction^c^	8772 (1.74)	19,073 (1.90)	0.0114	9310 (1.86)	18,598 (1.85)	0.0013
Stroke/TIA	20,558 (4.09)	48,586 (4.83)	0.0360	22,539 (4.51)	45,995 (4.57)	0.0025
Chronic kidney disease	102,444 (20.37)	242,093 (24.07)	0.0891	113,168 (22.66)	229,156 (22.75)	0.0021
Chronic liver disease	29,366 (5.84)	65,156 (6.48)	0.0266	31,508 (6.31)	63,193 (6.27)	0.0015
Chronic lung disease (asthma, COPD)	40,844 (8.12)	79,963 (7.95)	0.0063	40,789 (8.17)	81,048 (8.05)	0.0045
Chronic neurological disorders (Parkinson's)	4152 (0.83)	8974 (0.89)	0.0072	4292 (0.86)	8730 (0.87)	0.0008
Chronic sleep disorder^d^	105,850 (21.05)	200,088 (19.90)	0.0286	102,407 (20.51)	204,715 (20.32)	0.0045
Diabetes, type 1 or 2	121,446 (24.15)	286,585 (28.50)	0.0988	133,557 (26.74)	271,273 (26.93)	0.0042
Herpes zoster	13,680 (2.72)	17,768 (1.77)	0.0644	10,784 (2.16)	21,369 (2.12)	0.0026
HIV/AIDS	937 (0.19)	1257 (0.12)	0.0156	749 (0.15)	1468 (0.15)	0.0011
Obesity	121,093 (24.08)	287,290 (28.57)	0.1020	135,565 (27.14)	272,548 (27.06)	0.0020
Stem cell/solid organ transplant	2111 (0.42)	4851 (0.48)	0.0093	2390 (0.48)	4664 (0.46)	0.0023
Mental health conditions						
Anxiety	73,061 (14.53)	168,182 (16.72)	0.0604	80,557 (16.13)	161,360 (16.02)	0.0030
Depression	65,577 (13.04)	148,023 (14.72)	0.0485	71,846 (14.39)	143,081 (14.20)	0.0052
PTSD	2141 (0.43)	4793 (0.48)	0.0076	2310 (0.46)	4640 (0.46)	0.0003
Substance use disorder						
Alcohol use/abuse disorder	6378 (1.27)	16,060 (1.60)	0.0276	7363 (1.47)	14,957 (1.48)	0.0009
Nicotine dependence	21,147 (4.21)	73,074 (7.27)	0.1319	30,381 (6.08)	62,617 (6.22)	0.0055
Substance use/abuse disorder	6290 (1.25)	17,495 (1.74)	0.0403	7778 (1.56)	15,817 (1.57)	0.0010
**Medications**						
Anti‐infective medications	317,643 (63.17)	614,519 (61.10)	0.0426	311,072 (62.29)	623,684 (61.91)	0.0077
Antiviral medications	45,862 (9.12)	65,702 (6.53)	0.0964	37,812 (7.57)	75,311 (7.48)	0.0036
Autoimmune medications	31,940 (6.35)	50,543 (5.03)	0.0573	28,039 (5.61)	55,579 (5.52)	0.0042
Cardiovascular disease medications	418,235 (83.17)	834,546 (82.98)	0.0051	407,215 (81.54)	818,900 (81.29)	0.0063
Antihypertensives	344,318 (68.47)	714,694 (71.07)	0.0564	350,695 (70.22)	706,259 (70.11)	0.0024
Statins	303,482 (60.35)	567,533 (56.43)	0.0796	290,821 (58.23)	582,313 (57.81)	0.0086
Chronic kidney medication	197 (0.04)	458 (0.05)	0.0031	218 (0.04)	437 (0.04)	0.0001
COPD/asthma medication	128,840 (25.62)	246,727 (24.53)	0.0251	126,002 (25.23)	251,455 (24.96)	0.0062
Diabetes medication	87,431 (17.39)	198,569 (19.74)	0.0607	93,740 (18.77)	190,125 (18.87)	0.0027
Metformin	68,316 (13.59)	151,314 (15.05)	0.0417	72,238 (14.46)	146,150 (14.51)	0.0013
Sulfonylurea	23,745 (4.72)	62,675 (6.23)	0.0664	27,911 (5.59)	57,350 (5.69)	0.0045
HIV medication	1120 (0.22)	1495 (0.15)	0.0172	891 (0.18)	1758 (0.17)	0.0009
NSAID	117,877 (23.44)	232,657 (23.13)	0.0073	116,999 (23.43)	234,603 (23.29)	0.0033
Parkinson's	12,634 (2.51)	26,633 (2.65)	0.0086	13,145 (2.63)	26,319 (2.61)	0.0012
Psychotropic medication	175,624 (34.93)	346,346 (34.44)	0.0102	174,906 (35.02)	349,585 (34.70)	0.0067
Antipsychotics	7196 (1.43)	16,093 (1.60)	0.0138	7482 (1.50)	15,807 (1.57)	0.0063
Benzodiazepine	65,901 (13.11)	135,874 (13.51)	0.0119	67,206 (13.46)	134,987 (13.40)	0.0017
**Healthcare utilization**						
DME	142,530 (28.34)	292,940 (29.13)	0.0173	144,896 (29.01)	290,833 (28.87)	0.0031
ED visits						
Mean (SD)	0.28 (0.98)	0.39 (2.25)	0.0643	0.33 (1.25)	0.35 (2.02)	0.0117
0	414,281 (82.39)	798, 653 (79.41)	0.0757	401,545 (80.40)	810,071 (80.42)	0.0003
1	63,970 (12.72)	139,157 (13.84)	0.0329	67,320 (13.48)	135,679 (13.47)	0.0003
2+	24,594 (4.89)	67,880 (6.75)	0.0794	30,551 (6.12)	61,591 (6.11)	0.0001
Home health	22,536 (4.48)	64,289 (6.39)	0.0843	28,377 (5.68)	57,718 (5.73)	0.0021
Hospice	100 (0.02)	514 (0.05)	0.0166	180 (0.04)	398 (0.04)	0.0018
Hospitalizations						
Mean (SD)	0.12 (0.44)	0.18 (0.57)	0.1007	0.16 (0.51)	0.16 (0.54)	0.0061
0	455,207 (90.53)	883,213 (87.82)	0.0871	443,085 (88.72)	893,935 (88.74)	0.0007
1	37,257 (7.41)	88,860 (8.84)	0.0522	41,765 (8.36)	84,139 (8.35)	0.0004
2+	10,381 (2.06)	33,617 (3.34)	0.0789	14,565 (2.92)	29,266 (2.91)	0.0007
Outpatient encounters						
Mean (SD)	19.32 (15.68)	18.09 (15.36)	0.0795	18.64 (15.31)	18.61 (15.63)	0.0017
SNF	5156 (1.03)	17, 275 (1.72)	0.0596	7395 (1.48)	14,891 (1.48)	0.0002
Wellness visits	260,265 (51.76)	668,883 (66.51)	0.3035	308,082 (61.69)	620,502 (61.60)	0.0019
**Preventive vaccinations** ^e^						
Influenza	430,968 (85.71)	675,476 (67.17)	0.4477	369,729 (74.03)	739,933 (73.45)	0.0131
Pneumococcal	120,758 (24.01)	178,525 (17.75)	0.1546	102,009 (20.43)	200,533 (19.91)	0.0129
Tdap	43,458 (8.64)	29,698 (2.95)	0.2453	24,979 (5.00)	50,457 (5.01)	0.0003
ZVL	9476 (1.88)	15,404 (1.53)	0.0272	9008 (1.80)	16,833 (1.67)	0.0102

Abbreviations: ASMD, absolute standardized mean difference; COPD, chronic obstructive pulmonary disease; DME, durable medical equipment; ED, emergency department; HIV/AIDS, human immunodeficiency virus/acquired immune deficiency syndrome; IPTW, inverse probability of treatment weight; IQR, interquartile range; MI, myocardial infarction; NSAID, non‐steroidal anti‐inflammatory drug; PTSD, post‐traumatic stress disorder; RZV, recombinant zoster vaccine; SD, standard deviation; SNF, skilled nursing facility; Tdap, tetanus, diphtheria and pertussis; TIA, transient ischemic attack; ZVL, zoster live vaccine.

^a^ Race/ethnicity is self‐reported.

^b^ Other = Other or American Indian or Alaskan Native.

^c^ Cerebral infarction includes hemorrhage and traumatic brain injury.

^d^ Chronic sleep disorder includes narcolepsy, sleep apnea, insomnia.

^e^ All available data before the index date were used to identify zoster live vaccine and Tdap exposure.

Most demographic, chronic condition, medication, and healthcare utilization covariates had absolute SMDs < 0.10, before and after IPTW‐stabilized weighting. Initially, obesity, hypertension, nicotine dependence, wellness visits, hospitalizations, pneumococcal, Tdap, and influenza vaccinations were not balanced (absolute SMD > 0.10), but after IPTW‐stabilized weighting all SMDs were close to zero (Table 1).

### 3.2 Incidence of dementia

The average follow‐up time was 2.90 (±1.03) years for the RZV‐exposed and 2.35 (±1.23) years for the RZV‐unvaccinated comparators. Over the follow‐up, there were a total of 51,587 (3.42%) cases of dementia across the RZV‐exposed (*N* = 15,061; 3.00%) and the RZV‐unvaccinated comparators (*N* = 36,526; 3.63%). The average time to dementia was 1.85 (±1.03) years in the RZV‐exposed and 1.61 (±1.02) years in the RZV‐unvaccinated comparators. The incidence of dementia per 1000 person‐years was 10.45 (95% CI: 10.29, 10.62) for the RZV‐exposed and 15.73 (95% CI: 15.57, 15.89) for the RZV‐unvaccinated comparators (Table 2). Figure S2 illustrates the cumulative incidence of dementia by RZV‐exposed and RZV‐unvaccinated comparator. Table S4 shows the age‐ and sex‐stratified incidence of dementia, with increasing incidence with age but no difference by sex.

**TABLE 2:** Incidence rate and hazard ratio for new‐onset dementia, Alzheimer's disease, and vascular dementia among RZV‐exposed and RZV‐unvaccinated comparators.

Exposure group	Number of individuals	Events	PY	Incidence per 1000 PY	Follow‐up period	IPTW‐adjusted HR (95% CI)
**Dementia**						
RZV‐exposed	502,845	15,061	1,440,694	10.45 (10.29 to 10.62)	≤3 years	0.67 (0.65, 0.68)
					>3 years	0.74 (0.70, 0.79)
RZV‐unvaccinated	1,005,690	36,526	2,322,674	15.73 (15.57 to 15.89)	Reference	1.00
**Alzheimer's disease**						
RZV‐exposed	502,845	4314	1,454,156	2.97 (2.88 to 3.06)	≤3 years	0.72 (0.69, 0.74)
					>3 years	0.83 (0.75, 0.93)
RZV‐unvaccinated	1,005,690	9397	2,355,099	3.99 (3.91–4.07)	Reference	1.00
**Vascular dementia**						
RZV‐exposed	502,845	2161	1,457,065	1.48 (1.42 to 1.55)	≤3 years	0.67 (0.64, 0.70)
					>3 years	0.66 (0.57, 0.77)
RZV‐unvaccinated	1,005,690	5333	2,360,653	2.26 (2.20 to 2.32)	Reference	1.00

Abbreviations: CI, confidence interval; HR, hazard ratio; IPTW, inverse probability of treatment weight; PY, person years; RZV, recombinant zoster vaccine.

Notes: All, all follow‐up years; ≤3 and >3 years represent the time‐dependent coefficient of the interaction of exposure by time to account for violation of the proportional hazards assumption; models were adjusted for competing risk of death.

### 3.3 Incidence of AD and VD

The incidence of AD per 1000 person‐years was 2.97 (95% CI: 2.88, 3.06) for the RZV‐exposed and 3.99 (95% CI: 3.91, 4.07) for the RZV‐unvaccinated comparator (Table 2). The average time to AD for RZV‐exposed and RZV‐unvaccinated comparators was 2.00 (±1.03) years and 1.78 (±1.03) years, respectively. Figure S3 illustrates the cumulative incidence of AD for the RZV‐exposed and RZV‐unvaccinated comparator.

The incidence of VD per 1000 person‐years was 1.48 (95% CI: 1.42, 1.55) for the RZV‐exposed and 2.26 (95% CI: 2.20, 2.32) for the RZV‐unvaccinated comparator (Table 2). The average time to VD was similar to AD with 2.00 (±1.03) years and 1.78 (±1.04) years in the RZV‐exposed and RZV‐unvaccinated comparators, respectively. Figure S4 illustrates the cumulative incidence of VD for the RZV‐exposed and the RZV‐unvaccinated comparator.

### 3.4 Risk of dementia, AD, and VD

Table 2 presents the results from the IPTW‐stabilized weighted Cox proportional hazards models, adjusted for violation of the proportional hazards assumption using a time‐dependent coefficient as a piecewise constant that reflects the time‐treatment interaction. We selected 3 years based on the 3‐year mean follow‐up. New‐onset dementia with 3 years or less of follow‐up had HR = 0.67 (95% CI: 0.65, 0.68) and HR = 0.74 (95% CI: 0.69, 0.79) for more than 3 years of follow‐up. The risk of new‐onset AD with 3 years or less and more than 3 years of follow‐up was HR = 0.72 (95% CI: 0.69, 0.74) and HR = 0.83 (95% CI: 0.74, 0.94), respectively. The risk of new‐onset VD with 3 years or less and more than 3 years of follow‐up was HR = 0.67 (95% CI: 0.64, 0.70) and HR = 0.66 (95% CI: 0.57, 0.78), respectively. The HRs and 95% CIs, unadjusted for non‐proportionality, are presented in Table S5. Results from the stratified analyses reveal a similar risk across all age and sex strata (Table S4).

### 3.5 Sensitivity analyses

**FIGURE 2:**
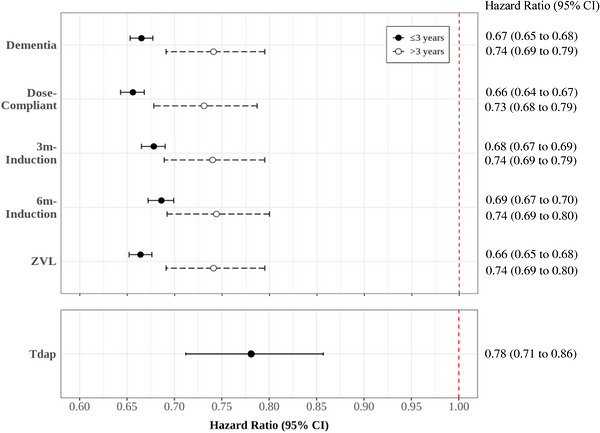
Hazard ratios for IPTW‐adjusted sensitivity analyses for the RZV‐exposed relative to RZV‐unvaccinated comparators. IPTW, inverse probability of treatment weight; CI, confidence interval; RZV, recombinant zoster vaccine; 3 m, 3‐month; 6 m, 6‐month; ZVL, zoster live vaccine; Tdap, tetanus, diphtheria, and pertussis. Time‐dependent coefficient, ≤3 years and >3 years, corrected for non‐proportional hazards. Tdap corresponds to the analysis within the subgroup with prior Tdap vaccination. ZVL corresponds to the analysis within the subgroup without prior ZVL vaccination.

The results of the sensitivity analyses, with adjustment for non‐proportional hazards, are summarized in Figure 2. The 90‐ and 183‐day induction periods generated an IPTW‐stabilized HR = 0.68 (95% CI: 0.67, 0.69) and HR = 0.69 (95% CI: 0.67, 0.70), respectively, for 3 years or less and HR = 0.74 (95% CI: 0.69, 0.79) and HR = 0.74 (95% CI: 0.69, 0.80), respectively, for more than 3 years of follow‐up. Despite a slightly higher point estimate, the 95% CIs overlapped with that of the primary analysis, suggesting that the primary analysis was robust to potential non‐differential outcome misclassification. The dose‐compliant and the ZVL sensitivity analyses, adjusted for non‐proportional hazards, demonstrated a lower risk of dementia for 3 years or less and more than 3 years of follow‐up (Figure 2). The sensitivity analysis in the subsample who received Tdap prior to the index date resulted in a risk estimate that was closer to the null relative to the primary analysis but maintained a statistically significant lower risk (HR = 0.78, 95% CI: 0.71, 0.86). The Tdap analysis did not violate the proportional hazards assumption, and IPTW‐stabilized results are presented in Figure 2.

The E‐value for the primary analysis showed that a risk ratio of at least 2.35 for the exposure–unmeasured confounder and unmeasured confounder–outcome association would be needed to shift the upper confidence interval to include the null (Figure S5).

## 4 DISCUSSION

Our study found a statistically significant lower risk of new‐onset dementia, AD, and VD with a two‐dose RZV regimen relative to a RZV‐unvaccinated comparator, after accounting for the competing risk of death. The results were consistent across all sensitivity analyses. The 26% to 33% lower risk of dementia following RZV reported in this study corresponds to the results from prior studies that did not distinguish among HZ vaccines.^10^, ^13^, ^14^ Two studies that evaluated HZ vaccines, but did not include RZV, reported HRs corresponding to a 22% to 28% lower risk of dementia.^13^, ^14^ A recent study using US commercial claims data reported a HR of 0.68 (95% CI: 0.67, 0.70) among those who received the RZV two‐dose regimen.^27^ While a significant reduction in the risk of dementia was reported for those who received a single RZV dose (HR = 0.89; 95% CI: 0.87, 0.92), the effect was not as strong as the two‐dose regimen.^27^ Despite the slightly younger cohort in the Tang et al.^27^ study relative to the present study, the risk estimates for the two‐dose RZV exposure in their study were similar to ours. The 2‐ to 6‐month dose‐compliant sensitivity analysis showed a statistically significant lower risk of dementia similar to the primary analysis. Moreover, the present study findings align with studies in Wales and Australia that reported a statistically significant reduction in new‐onset dementia among adults ≥70 years old following live‐attenuated HZ vaccine.^18^, ^19^

The data provide consistent evidence of a significantly lower risk of dementia, including AD and VD, following the two‐dose RZV vaccination. Our findings align with several studies that demonstrate a lower risk of AD and VD following HZ vaccination. Scherrer and colleagues reported a lower risk of AD among US veterans (HR = 0.75 [95% CI:0.71, 0.80]) and those with commercial insurance (HR = 0.70 [95% CI: 0.55, 0.88]) following HZ vaccination.^10^ A study using the Clinical Practice Research Datalink representing UK citizens reported an AD risk reduction following HZ vaccination.^13^ A lower risk of AD and VD following HZ vaccination was also found in a Welsh population (HR = 0.81; 95% CI: 0.77, 0.86 and HR = 0.66; 95% CI: 0.61, 0.71, respectively).^14^

Potential mechanisms of the lower risk of dementia following RZV vaccination may be due to the mitigation of neurotropic infection burden and trained immunity.^28^ Chronic infection with the herpes viruses can be a risk factor for dementia due to the deregulation of transposable elements in the aging brain.^29^

Dementia prevalence is expected to triple in the next 50 years.^2^ Consequently, healthcare utilization and financial burden associated with the onset of dementia in adults aged 55 years and older will be substantial.^30^ In this study, RZV was associated with a reduction in the risk of dementia by potentially delaying its onset, which we found to be approximately 3 months, on average. This is similar to the findings in other studies.^17^ Delaying the onset of dementia can alleviate stress on families and caregivers, reduce strain on healthcare resources, and improve the quality of life among older adults.

This study has several notable strengths. For one, we included a large, population‐based sample of US adults aged 65 years or older. The matched cohort mitigated imbalances in age, sex, and race/ethnicity between RZV‐exposed and the RZV‐unvaccinated comparators. We implemented several sensitivity analyses to test the robustness of the primary analysis. Adjustment for a comprehensive list of covariates mitigated confounding. This is one of few studies to adjust for several common adult vaccines.

Despite the study's strengths, several of its limitations warrant consideration. There is a potential for outcome misclassification since we could not validate the diagnoses with the medical chart. We cannot rule out potential confounding by educational attainment, marital status, and physical activity, which we could not measure. Medicare fee‐for‐service disenrollment may have limited detection of incident dementia. Most individuals qualify for Medicare at age 65, and because we required a 365‐day baseline enrollment in Medicare, our cohort may underrepresent individuals aged 65. We do not expect appreciable differences in dementia risk between those excluded due to Medicare Part C (i.e., Advantage plans) and Medicare fee‐for‐service beneficiaries. The analytic cohorts overrepresent Whites, relative to 76.6% Whites in the US Census aged 65 or older^31^ or the 76% to 82% Whites enrolled in Medicare fee‐for‐service,^32^, ^33^ which may reflect racial differences in the use of preventive services.^34^, ^35^ The matched RZV‐exposed and the RZV‐unvaccinated comparators mitigate the potential for confounding by race/ethnicity, age, and sex but also limited our ability to detect subgroup differences in dementia risk. Our quantitative bias analysis suggested that an unmeasured confounder with a relative risk >2, above what has already been controlled for, would be needed to explain away the effect. Given the extensive list of confounders accounted for in this study, it is not likely that such an unmeasured confounder exists. Individuals who self‐select to receive both RZV doses may have introduced selection bias toward healthy individuals. To mitigate healthy‐user bias, we identified comparators who had a preventive care visit. The study design and analytic methods were carefully constructed to ensure scientific rigor in the design, confounder adjustment methods, and sensitivity analyses, but random sources of error cannot be ruled out in this observational study.

### 4.1 Conclusion

This large population‐based comparator cohort study found a significantly lower risk of new‐onset dementia, AD, and VD following two‐dose RZV vaccination. The finding was robust to several sensitivity analyses that accounted for disease latency and prior Tdap and ZVL exposure. Disentangling the effect of different preventive vaccines on the reduction in the risk of dementia onset is an important area for future research. Replication of the findings with a longer follow‐up is warranted.

## CONFLICT OF INTEREST STATEMENT

SdR receives funding from GSK, the National Institutes of Health, the Patient Centered Outcomes Research Institute, Genentech, and consults for Alexion. H.A., D.O., and H.Y. are GSK employees. H.A., D.O., and H.Y. hold financial equities in GSK. All the other authors have no conflicts to disclose. The authors declare no other financial or non‐financial relationships and activities. Author disclosures are available in the Supporting Information.

## CONSENT STATEMENT

The University of Maryland Baltimore Institutional Review Board approved the study. The study was deemed exempt with a waiver of consent since there was no direct contact with individuals.

## Supporting information



Supporting Information

Supporting Information
