# Supplementary material for: Reduced risk of dementia with recombinant zoster vaccine in US adults age 65 or older
Source: Alzheimers Dement. 2026 Apr 28;22(5):e71407. doi: 10.1002/alz.71407 (PMC13124665; doi:10.1002/alz.71407)
Supplement: Supplementary file 2 — Supporting Information [file ALZ-22-e71407-s001.docx]

**Supplemental Material**

Tables

Table S1. List of preventive care visits and codes

Table S2. Outcome case ascertainment algorithms

Table S3: Description of the baseline covariates and data collection timeframe

Table S4. Age and sex-stratified incidence rate and hazard ratio for new-onset dementia

Table S5. Incidence rate and hazard ratio for new-onset dementia, AD, and VD among RZV-exposed and RZV-unvaccinated comparators; unadjusted for non-proportional hazards

Figures

Figure S1: Illustration of the cohort study design

Figure S2: Cumulative incidence of dementia for RZV-exposed and RZV-unvaccinated comparators

Figure S3: Cumulative incidence of AD for RZV-exposed and RZV-unvaccinated comparators

Figure S4: Cumulative incidence of VD for RZV-exposed and RZV-unvaccinated comparators

Figure S5: E-value for a weighted hazard of dementia

**Table S1: List of preventive care visit codes**

| **Preventive Care Visit Description** | **Code** | **Source** | **Type** |
| --- | --- | --- | --- |
| **Routine Adult Annual Exam** |  |  |  |
| Initial comprehensive preventive medicine evaluation and management of an individual including an age and gender appropriate history, examination, counseling/anticipatory guidance/risk factor reduction interventions, and the ordering of laboratory/diagnostic procedures, new patient; 40-64 years | 99386 | CPT-4 | Procedure |
| Initial comprehensive preventive medicine evaluation and management of an individual including an age and gender appropriate history, examination, counseling/anticipatory guidance/risk factor reduction interventions, and the ordering of laboratory/diagnostic procedures, new patient; 65 years and older | 99387 | CPT-4 | Procedure |
| Periodic comprehensive preventive medicine reevaluation and management of an individual including an age and gender appropriate history, examination, counseling/anticipatory guidance/risk factor reduction interventions, and the ordering of laboratory/diagnostic procedures, established patient; 40-64 years | 99396 | CPT-4 | Procedure |
| Periodic comprehensive preventive medicine reevaluation and management of an individual including an age and gender appropriate history, examination, counseling/anticipatory guidance/risk factor reduction interventions, and the ordering of laboratory/diagnostic procedures, established patient; 65 years and older | 99397 | CPT-4 | Procedure |
| Annual wellness visit; includes a personalized prevention plan of service (PPS), initial visit | G0438 | HCPCS | Procedure |
| Annual wellness visit, includes a personalized prevention plan of service (PPS), subsequent visit | G0439 | HCPCS | Procedure |
| Federally qualified health center (FQHC) visit that includes an initial preventive physical examination (IPPE) or annual wellness visit (AWV) and includes a typical bundle of Medicare-covered services that would be furnished per diem to a patient receiving an IPPE or AWV | G0468 | HCPCS | Procedure |
| Wellness assessment, performed by non-physician | S5190 | HCPCS | Procedure |
| Initial preventive physical examination; face-to-face visit, services limited to new beneficiary during the first 12 months of Medicare enrollment | G0402 | HCPCS | Procedure |
| Encounter for general adult medical exam without abnormal findings | Z00.00 | ICD-10-CM | Diagnosis |
| Encounter for general adult medical examination with abnormal findings | Z00.01 | ICD-10-CM | Diagnosis |
| **Colonoscopy Preventive Screening Intervention** |  |  |  |
| Colonoscopy, flexible; diagnostic, including collection of specimen(s) by brushing or washing, when performed (separate procedure) | 45378 | CPT-4 | Procedure |
| Colorectal cancer screening; colonoscopy on individual at high risk | G0105 | HCPCS | Procedure |
| Colorectal cancer screening; colonoscopy on individual not meeting criteria for high risk | G0121 | HCPCS | Procedure |
| Patients greater than 85 years of age who received a routine colonoscopy for a reason other than the following: an assessment of signs/symptoms of gi tract illness, and/or the patient is considered high risk, and/or to follow-up on previously diagnosed advance lesions | G9661 | HCPCS | Procedure |
| Patients greater than 85 years of age who did not have a history of colorectal cancer or valid medical reason for the colonoscopy, including: iron deficiency anemia, lower gastrointestinal bleeding, Crohn's disease (i.e., regional enteritis), familial adenomatous polyposis, lynch syndrome (i.e., hereditary non-polyposis colorectal cancer), inflammatory bowel disease, ulcerative colitis, abnormal finding of gastrointestinal tract, or changes in bowel habits | G9659 | HCPCS | Procedure |
| Ca screen; flexi sigmoidscope | G0104 | HCPCS | Procedure |
| colorectal screening ca screen; barium enema | G0106 | HCPCS | Procedure |
| colorectal screening RECTAL CANCER SCREENING; FECAL-OCCULT BLOOD TEST | G0107 | HCPCS | Procedure |
| colorectal screening ca screen; barium enema | G0120 | HCPCS | Procedure |
| colorectal screening ca screen; barium enema | G0122 | HCPCS | Procedure |
| colorectal screening rectal cancer screening, fecal occult blood test, | G0328 | HCPCS | Procedure |
| Encounter for screening for malignant neoplasm of colon | Z12.11 | ICD-10-CM | Diagnosis |
| **Mammography Preventive Screening Intervention** |  |  |  |
| Screening mammography, bilateral (two-view study of each breast), including computer-aided detection (CAD) when performed | 77067 | CPT-4 | Procedure |
| Screening mammography, bilateral (two-view film study of each breast) Office/Freestanding (Global) | 77057 | CPT-4 | Procedure |
| Screening mammography, bilateral (two view film study of each breast) | 76092 | CPT-4 | Procedure |
| Screening mammography, bilateral (two-view study of each breast), including computer- aided detection (CAD) when performed | G0202 | HCPCS | Procedure |
| Diagnostic mammography, including computer-aided detection (CAD) when performed; bilateral | G0203 | HCPCS | Procedure |
| Screening mammogram for high-risk patient | V7611 | HCPCS | Procedure |
| Encounter for screening mammogram for malignant neoplasm of breast | V7612 | HCPCS | Procedure |
| Diagnostic mammography, including computer-aided detection (CAD) when performed; bilateral | G0204 | HCPCS | Procedure |
| Diagnostic mammography, including computer-aided detection (CAD) when performed; unilateral | G0206 | HCPCS | Procedure |
| Encounter for screening for malignant neoplasm of breast | Z12.3 | ICD-10-CM | Diagnosis |
| Encounter for screening mammogram for malignant neoplasm of breast | Z12.31 | ICD-10-CM | Diagnosis |
| Encounter for other screening for malignant neoplasm of breast | Z12.39 | ICD-10-CM | Diagnosis |
| **Osteoporosis Preventive Screening Interventions** |  |  |  |
| Dual-energy X-ray absorptiometry (DXA), bone density study, 1 or more sites; axial skeleton (e.g., hips, pelvis, spine) | 77080 | CPT-4 | Procedure |
| Dual-energy X-ray absorptiometry (DXA), bone density study, 1 or more sites; appendicular skeleton (peripheral) (e.g., radius, wrist, heel) | 77081 | CPT-4 | Procedure |
| PERIPHERAL SKELETAL BONE MINERAL DENSITYN20030626 | G0062 | HCPCS | Procedure |
| CENTRAL SKELETAL BONE MINERAL DENSITY STN20030626 | G0063 | HCPCS | Procedure |
| SINGLE ENERGY X-RAY ABSORPTIOMETRY (SEXAN20110101: Single energy x-ray absorptiometry (sexa) bone density study, one or more sites; appendicular skeleton (peripheral) (e.g., radius, wrist, heel) (Single energy x-ray study) | G0130 | HCPCS | Procedure |
| COMPUTERIZED TOMOGRAPHY BONE MINERAL DENN20060101 | G0131 | HCPCS | Procedure |
| ULTRA-SOUND BONE MINERAL STUDY, ONE OR MORE SITES, APPENDICULAR SKELETON | G0132 | HCPCS | Procedure |
| Bone mineral density | G0133 | HCPCS | Procedure |
| Encounter screening for osteoporosis | Z13.820 | ICD-10-CM | Diagnosis |

CPT, current procedural terminology; HCPCS, healthcare common procedure coding system; ICD, international classification of diseases.

**Table S2. Outcome case ascertainment algorithms**

| **Outcome** | **ICD-10 Codes** | **Validation Statistics^a^** | **Other Requirements^b^** | **Setting for Case Ascertainment^c,d^** | **Follow-up Period** | **Look-back Period for Incidence** |
| --- | --- | --- | --- | --- | --- | --- |
| Dementia  (primary) | F01.5x, F01.Ax, F01.Bx, F01.Cx, F02.8x, F02.Ax, F02.Bx, F02.Cx, F03.9x, F03.Ax, F03.Bx, F03.Cx, G30.0, G30.1, G30.8, G30.9, G31.0, G31.01, G31.09, G31.1, G31.83 | 1-year  Sensitivity:  64% (56%, 72%)^1^  31.3% (27.5%, 35%)^2^  Specificity:  93% (92%, 95%)^1^  98% (97.7%, 98.4%)^2^  PPV:  58% (51%, 66%)^1^  70.3% (65%, 75.6%)^2^ | CCW chronic condition code does not indicate history of AD or non-AD dementia | >1 inpatient, SNF, HHA, hospice, or DME claim, OR >2 outpatient or carrier (provider) claim,  any position & > 7 days apart, OR 1 outpatient or carrier claim and 1 inpatient, SNF, HHA, hospice, or DME claim at least 1 day apart | Day 1 post-index until earliest of HOI or a censoring event | 1 year from index date, all settings, for dementia, vascular dementia, AD, MCI |
| VD  (secondary) | F01.5x, F01.Ax, F01.Bx, F01.Cx |  | ··· | ··· | ··· | ··· |
| AD  (secondary) | G30.0, G30.1, G30.8, G30.9 |  | ··· | ··· | ··· | ··· |

AD, Alzheimer’s Disease; CCW, chronic condition warehouse; DME, Durable Medical Equipment; HHA, Home Health Agency; HOI, health outcome of interest; MCI, mild cognitive impairment; PPV, positive predictive value SNF, Skilled Nursing Facility; VD, vascular dementia.

··· , same as the information for dementia.

^a^ Validation statistics are based on the Bynam-Standard 1-year for dementia; not applicable for secondary/exploratory outcomes.

^b^ CCW chronic condition code does not indicate history of AD or non-AD dementia.

^c^ Bynam-standard algorithm to avoid misclassification of the outcome due to the potential for 1 visit to reflect rule-out diagnosis. The hospital outpatient file captures underserved populations receiving care from Federally Qualified Health Clinics, rural health centers, or critical access hospitals, which appear in the outpatient file.

^d^ Includes inpatient, SNF, HHA, hospital outpatient, carrier (physician) settings.

^1^ Grodstein, F., et al., Identification of dementia in recent Medicare claims data, compared with rigorous clinical assessments. J Gerontol A Biol Sci Med Sci. 2022; 77(6):1272-78.

^2^ McCarthy, E.P., et al. Validation of claims algorithms to identify Alzheimer’s Disease and Related Dementias. J Gerontol A Biol Sci Med Sci. 2022; 77(6):1261-1271.

**Table S3. Description of the baseline covariates and data collection timeframe**

| Variable Type | Variable | Variable Measure | Collection Timeframe |
| --- | --- | --- | --- |
| Demographic | Age | categorical | At index date |
|  | Sex | binary | At index date |
|  | Race/ethnicity | categorical | At index date |
|  | US Region | categorical | At index date |
|  | RZV Dose 2 Year/calendar month & year | numerical | At index date |
| Chronic Medical Conditions | Diabetes – type 1 or 2 | binary | baseline |
|  | Obesity | binary | baseline |
|  | Hypertension | binary | baseline |
|  | Stroke/transient ischemic attack | binary | baseline |
|  | Ischemic heart disease/myocardial infarction | binary | baseline |
|  | Congestive heart failure | binary | baseline |
|  | Cardiovascular disease | binary | baseline |
|  | Chronic lung disease (asthma, chronic obstructive pulmonary disease) | binary | baseline |
|  | Cerebrovascular, including cerebral infarction, hemorrhage, and traumatic brain injury | binary | baseline |
|  | Herpes zoster | binary | baseline |
|  | Chronic kidney disease | binary | baseline |
|  | Chronic liver disease | binary | baseline |
|  | HIV/AIDS | binary | baseline |
|  | Cancer/chemotherapy | binary | baseline |
|  | Stem cell transplant/solid organ transplant | binary | baseline |
|  | Multiple sclerosis | binary | baseline |
|  | Crohn's disease | binary | baseline |
|  | Psoriasis/Psoriatic Arthritis | binary | baseline |
|  | Lupus | binary | baseline |
|  | Rheumatoid arthritis | binary | baseline |
|  | Graves’ Disease | binary | baseline |
|  | Vasculitis | binary | baseline |
|  | Neurological disorders (Parkinson’s) | binary | baseline |
|  | Sleep disorders | binary | baseline |
| Mental Health Condition | Depression | binary | baseline |
|  | Anxiety | binary | baseline |
|  | Post-traumatic stress disorders | binary | baseline |
| Substance Use Disorder | Nicotine dependence | binary | baseline |
|  | Alcohol use/abuse disorder | binary | baseline |
|  | Substance use/abuse disorder | binary | baseline |
| Medications | Anticholinergics | binary | baseline |
|  | Non-steroidal anti-inflammatory drugs | binary | baseline |
|  | Parkinson’s | binary | baseline |
|  | Cardiovascular disease | binary | baseline |
|  | Antihypertensives | binary | baseline |
|  | Statins | binary | baseline |
|  | Steroids | binary | baseline |
|  | Anti-infective medications | binary | baseline |
|  | Antiviral medications | binary | baseline |
|  | Metformin | binary | baseline |
|  | Sulfonylurea | binary | baseline |
|  | Diabetes medication | binary | baseline |
|  | Psychotropic medication | binary | baseline |
|  | HIV medication | binary | baseline |
| Healthcare Utilization | Outpatient encounters | number of outpatient encounters | baseline |
|  | Well visits | number of well visits | baseline |
|  | Durable medical equipment | binary | baseline |
|  | Hospitalizations | number in past year – as 0, 1, 2+ | baseline |
|  | Emergency department visits | number in past year – as 0, 1, 2+ | baseline |
|  | Specialized nursing facility | binary | baseline |
|  | Home health | binary | baseline |
| Vaccinations | Zostavax->12mos | Binary | baseline->365 days/ all available data |
|  | Zostavax-post | Date | follow-up censor |
|  | Tetanus, diphtheria, & pertussis | Binary | Baseline / all available data |
|  | Pneumococcal | Binary | baseline |
|  | Influenza | Binary | baseline |

HIV/AIDS, human immunodeficiency virus/acquired immunodeficiency syndrome; RZV, recombinant zoster vaccine.

**Table S4. Age and sex-stratified incidence rate and hazard ratio for new-onset dementia**

|  | **Exposure Group** | **Number of Individuals** | **Number of Events** | **Person Years** | **Incidence per 1000 PY** | **Crude HR (95%CI)** | **Covariate Adjusted HR (95% CI)** | **IPTW Adjusted HR (95% CI)** | |
| --- | --- | --- | --- | --- | --- | --- | --- | --- | --- |
| **Age Strata** |  |  |  |  |  |  |  |  | |
| Age 65–69 | RZV | 137,230 | 1,104 | 381,382 | 2.89  (2.73 – 3.07) | 0.64  (0.60 – 0.68) | 0.77  (0.71 – 0.84) | 0.68  (0.64 – 0.73) | |
|  | Comparator | 275,907 | 2,804 | 628,301 | 4.46  (4.30 – 4.63) |  |  |  | |
| Age 70–74 | RZV | 161,882 | 2,631 | 474,952 | 5.54  (5.33 – 5.76) | 0.62  (0.60 – 0.65) | 0.74  (0.70 – 0.78) | 0.66  (0.64 – 0.69) | |
|  | Comparator | 322,317 | 6,482 | 754,624 | 8.59  (8.38 – 8.80) |  |  |  | |
| Age 75–79 | RZV | 113,024 | 3,832 | 331,487 | 11.56  (11.20 – 11.93) | 0.66  (0.63– 0.68) | 0.72  (0.69 – 0.75) | 0.68  (0.66 – 0.71) | |
|  | Comparator | 226,261 | 9,030 | 528,803 | 17.08  (16.73 – 17.43) |  |  |  | |
| Age 80–84 | RZV | 58,267 | 3,577 | 166,227 | 21.52  (20.82 – 22.24) | 0.64  (0.62– 0.66) | 0.69  (0.66 – 0.72) | 0.66  (0.64 – 0.69) | |
|  | Comparator | 116,321 | 8,600 | 268,257 | 32.06  (31.39 – 32.74) |  |  |  | |
| Age 85+ | RZV | 32,442 | 3,917 | 86,646 | 45.21  (43.81 – 46.65) | 0.65  (0.63– 0.67) | 0.71  (0.69 – 0.74) | 0.67  (0.65 –0.70) | |
|  | Comparator | 64,884 | 9,610 | 142,690 | 67.35  (66.02 – 68.71) |  |  |  | |
| **Sex Strata** |  |  |  |  |  |  |  |  | |
| Male | RZV | 203,577 | 6,274 | 583,481 | 10.75  (10.49 – 11.02) | 0.65  (0.64 – 0.67) | 0.71  (0.69 – 0.74) | 0.67  (0.66 – 0.69) | |
|  | Comparator | 407,154 | 14,865 | 936,162 | 15.88  (15.63 – 16.14) |  |  |  | |
| Female | RZV | 299,268 | 8,787 | 857,213 | 10.25  (10.04 – 10.47) | 0.64  (0.63 – 0.65) | 0.72  (0.70 – 0.73) | 0.67  (0.66 – 0.69) | |
|  | Comparator | 598,536 | 21,661 | 1,386,512 | 15.62  (15.42 – 15.83) |  |  |  | |
| PY, person-years; HR, hazard ratio; CI, confidence interval; IPTW, inverse probability of treatment weight; RZV, recombinant zoster vaccine.  Covariate adjusted included all baseline covariates except for the matching variables (age, sex, race, index year).  The weighted Cox model was conducted using stabilized IPTW weights. | | | | | | | | |  |

**Table S5: Incidence rate and hazard ratio for new-onset dementia, Alzheimer’s Disease, and Vascular Dementia among RZV-exposed and RZV**-**unvaccinated comparators; unadjusted for non-proportional hazards**

| Exposure Group | Number of Individuals | Events | PY | Incidence per 1000 PY |  | IPTW Adjusted HR (95% CI) |
| --- | --- | --- | --- | --- | --- | --- |
| Dementia | | | | | | |
| RZV-exposed | 502,845 | 15,061 | 1,440,694 | 10.45  (10.29–10.62) |  | 0.67 (0.66–0.68)​ |
| RZV-unvaccinated | 1,005,690 | 36,526 | 2,322,674 | 15.73  (15.57–15.89) | Reference | 1.00 |
| Alzheimer’s Disease | | | | | | |
| RZV-exposed | 502,845 | 4,314 | 1,454,156 | 2.97  (2.88–3.06) |  | 0.73 (0.71–0.76)​ |
| RZV-unvaccinated | 1,005,690 | 9,397 | 2,355,099 | 3.99  (3.91–4.07) | Reference | 1.00 |
| Vascular Dementia | | | | | | |
| RZV-exposed | 502,845 | 2,161 | 1,457,065 | 1.48  (1.42–1.55) |  | 0.67 (0.64–0.70)​ |
| RZV-unvaccinated | 1,005,690 | 5,333 | 2,360,653 | 2.26  (2.20–2.32) | Reference | 1.00 |

CI, confidence interval; HR, hazard ratio; IPTW, inverse probability of treatment weight; PY, person years; RZV, recombinant zoster vaccine.


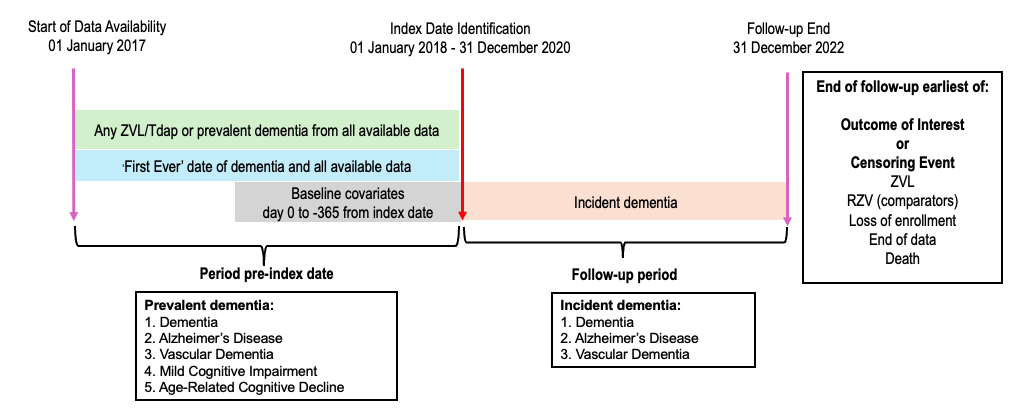
Figure S1: Illustration of the cohort study design

RZV, recombinant zoster vaccine; Tdap, tetanus, diphtheria, and pertussis; ZVL, zoster live vaccine.

Index date identification was the date of RZV dose 2 for RZV-exposed and the preventive care visit date for the RZV-unvaccinated; RZV-exposed and RZV-unvaccinated were matched on two-year age bands, sex, race/ethnicity within three-month intervals from 2018–2020.

‘First Ever’ date is a CCW chronic condition variable that identifies the first ever diagnosis in the individual’s history. Data are only available from 2017 onward, but the ‘First Ever’ date of diagnosis recorded in the data may be before 2017. Any dementia from all available data or the ‘First Ever’ date that occurs before or on the index date was considered prevalent dementia and not included in the cohort.

Figure S2: Cumulative incidence of dementia for RZV-exposed and RZV-unvaccinated comparators, unweighted


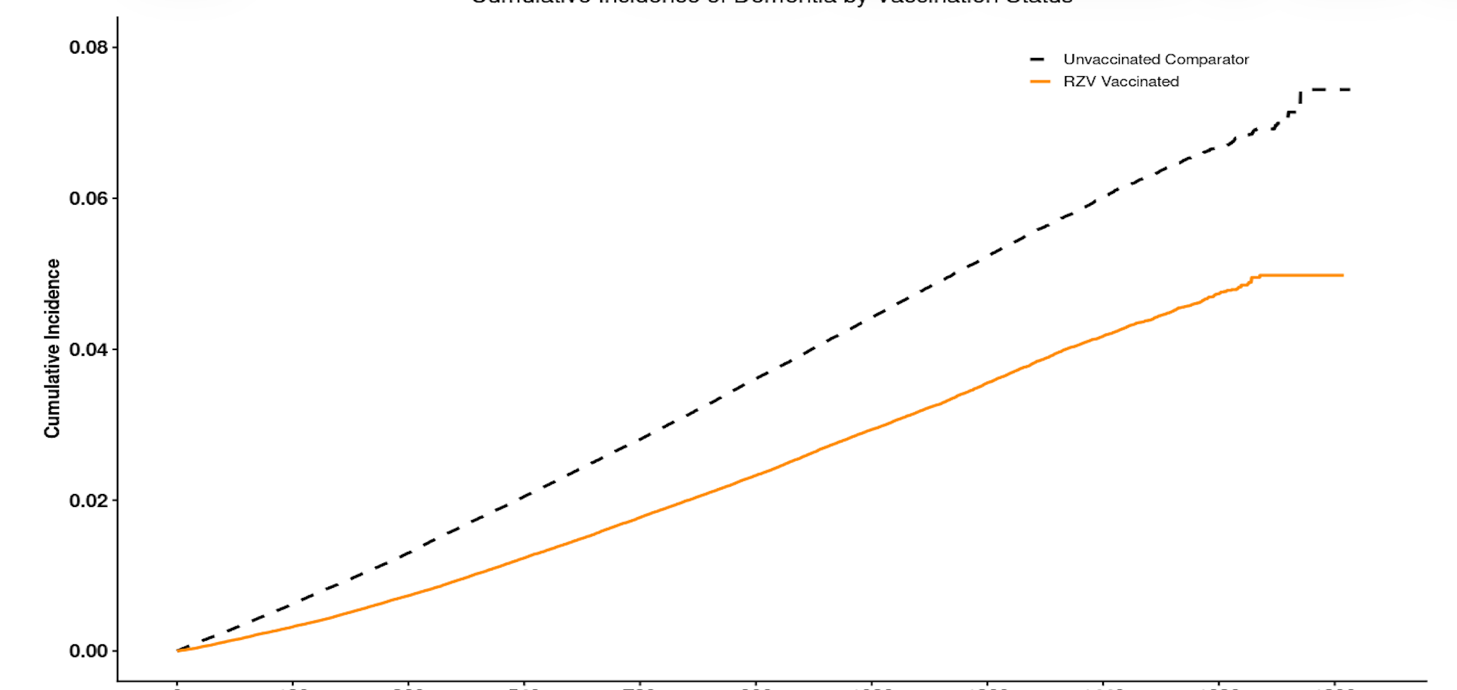

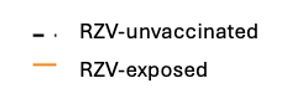

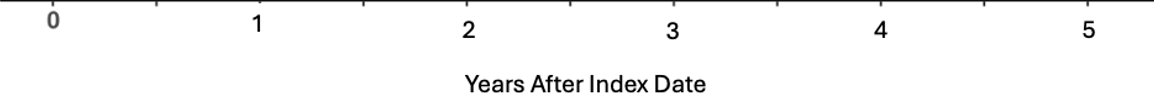


|  | Years After Index Date | | | | | | |  |
| --- | --- | --- | --- | --- | --- | --- | --- | --- |
| Dementia |  | 0 | 1 | 2 | 3 | 4 | 5 |  |
| RZV-exposed | At risk | 502,845 | 467,306 | 435,257 | 268,399 | 79,308 | <11 |  |
|  | Events | 0 | 3,562 | 8,369 | 12,603 | 14,752 | 15,061 |  |
|  | Incidence | 0 | 0.0073 | 0.0177 | 0.0294 | 0.0417 | 0.0498 |  |
| RZV-unvaccinated | At risk | 1,005,690 | 808,223 | 668,330 | 360,088 | 92,378 | 90 |  |
|  | Events | 0 | 11,786 | 23,392 | 32,279 | 36,035 | 36,526 |  |
|  | Incidence | 0 | 0.0130 | 0.0280 | 0.0442 | 0.0601 | 0.0744 |  |
| Note: Events and incidence represent cumulative number of events and cumulative incidence over time. RZV, recombinant zoster vaccine; adjusted for competing risk of death. | | | | | | | | |

**Figure S3**: **Cumulative incidence of AD for RZV-exposed and RZV-unvaccinated comparators, unweighted**


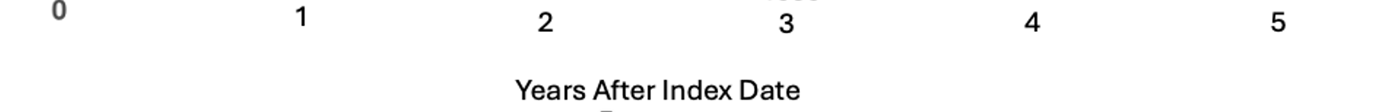

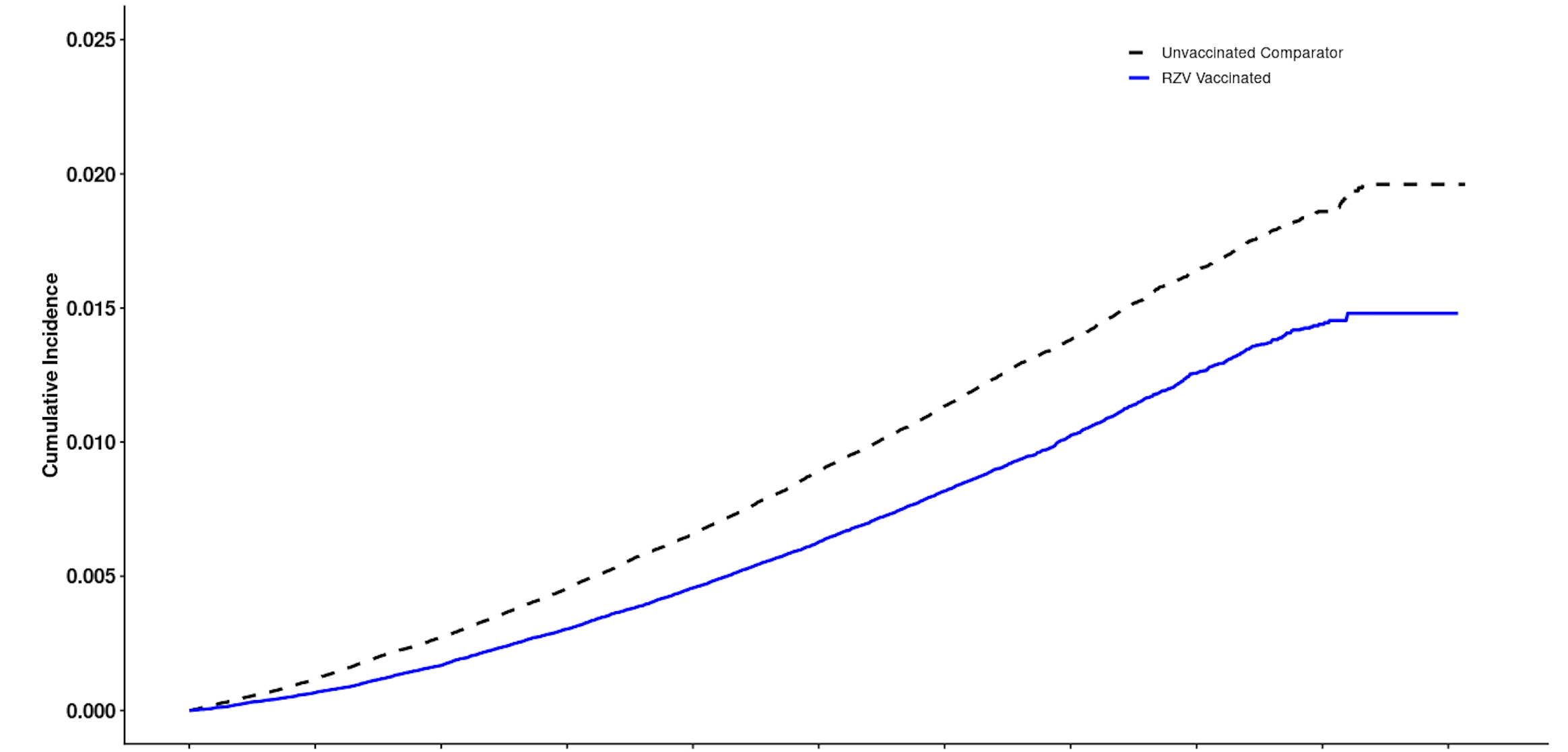

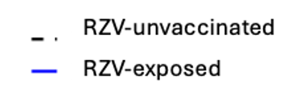


|  | Years After Index Date | | | | | | |  |
| --- | --- | --- | --- | --- | --- | --- | --- | --- |
| Alzheimer’s Disease |  | 0 | 1 | 2 | 3 | 4 | 5 |  |
| RZV-exposed | At risk | 502,845 | 469,678 | 440,068 | 273,211 | 81,309 | <11 |  |
|  | Events | 0 | 8,12 | 2,152 | 3,461 | 4,209 | 4,314 |  |
|  | Incidence | 0 | 0.0017 | 0.0046 | 0.0082 | 0.0126 | 0.0148 |  |
| RZV-unvaccinated | At risk | 1,005,690 | 815,599 | 680,309 | 370,084 | 95,895 | 95 |  |
|  | Events | 0 | 2,456 | 5,424 | 8,037 | 9,232 | 9,397 |  |
|  | Incidence | 0 | 0.0027 | 0.0066 | 0.0113 | 0.0164 | 0.0196 |  |
| Note: Events and incidence represent cumulative number of events and cumulative incidence over time. AD, Alzheimer’s Disease; RZV, recombinant zoster vaccine; adjusted for competing risk of death. | | | | | | | |  |

Figure S4: Cumulative incidence of VD for RZV-exposed and RZV-unvaccinated comparators, unweighted


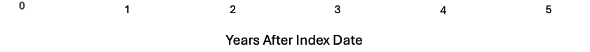

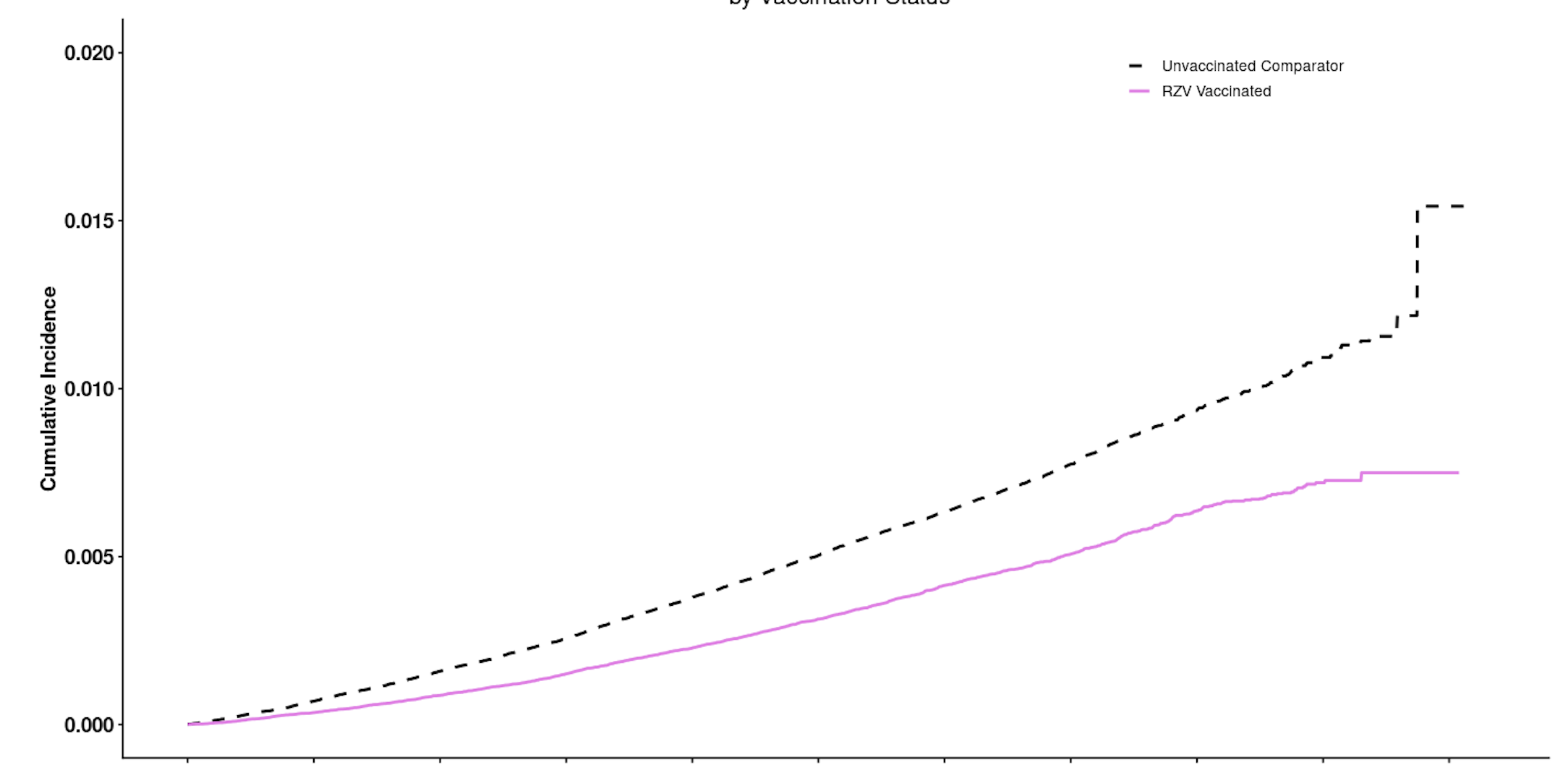

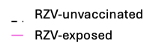


|  | Years After Index Date | | | | | | |  |
| --- | --- | --- | --- | --- | --- | --- | --- | --- |
| Vascular Dementia |  | 0 | 1 | 2 | 3 | 4 | 5 |  |
| RZV-exposed | At risk | 502,845 | 470,049 | 441,060 | 274,298 | 81,886 | <11 |  |
|  | Events | 0 | 419 | 1,072 | 1,746 | 2,115 | 2,161 |  |
|  | Incidence | 0 | 0.0009 | 0.0023 | 0.0041 | 0.0064 | 0.0075 |  |
| RZV-unvaccinated | At risk | 1,005,690 | 816,525 | 682,274 | 372,111 | 96,694 | 95 |  |
|  | Events | 0 | 1,430 | 3,115 | 4,520 | 5,222 | 5,333 |  |
|  | Incidence | 0 | 0.0016 | 0.0038 | 0.0063 | 0.0093 | 0.0154 |  |
| Note: Events and incidence represent cumulative number of events and cumulative incidence over time. RZV, recombinant zoster vaccine; VD, vascular dementia; adjusted for competing risk of death. | | | | | | | | |

**Figure S5: E-value for a weighted hazard of dementia**


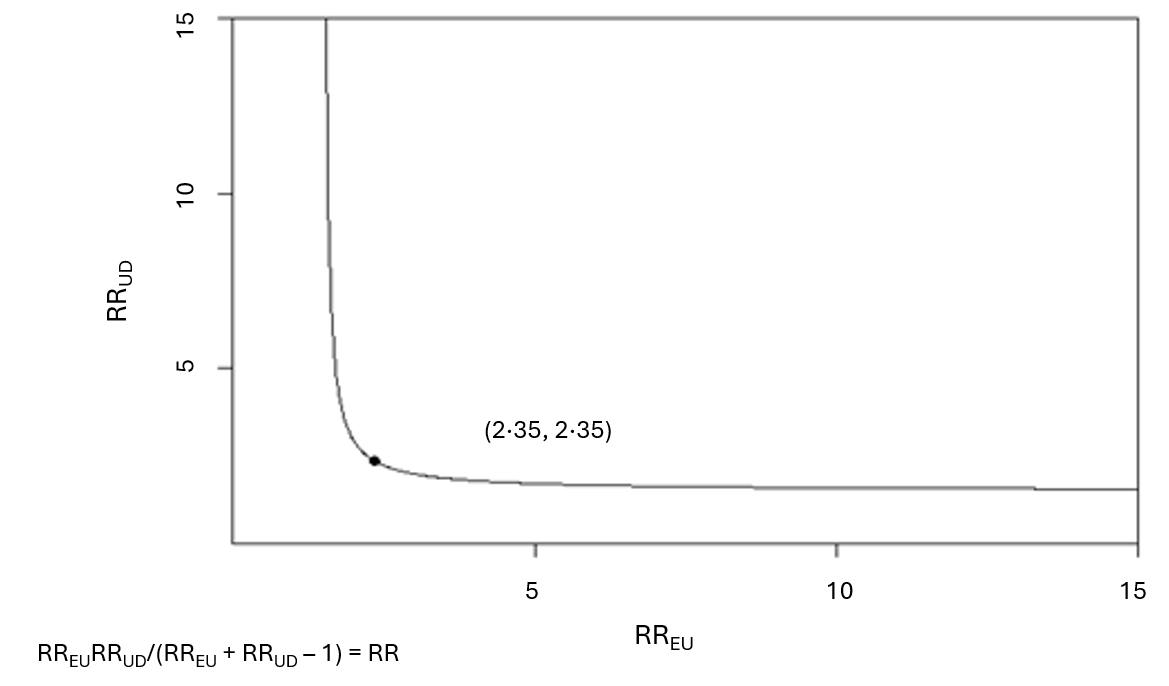


The E-value assessment was based on a weighted hazard ratio of 0.67 (95% confidence interval: 0.66–0.68).

ED, exposure-unmeasured confounder; RR, relative risk; UD, unmeasured confounder-outcome.
